# Supplementary material for: Bovine Serum Albumin Molecularly Imprinted Electrochemical Sensors Modified by Carboxylated Multi-Walled Carbon Nanotubes/CaAlg Hydrogels
Source: Gels. 2023 Aug 20;9(8):673. doi: 10.3390/gels9080673 (PMC10454541; doi:10.3390/gels9080673)
Supplement: Supplementary file 1 [file gels-09-00673-s001.zip › gels-2505167-supplementary.pdf]

Supplementary material

BSA molecularly imprinted electrochemical sensor modified by carboxylated multi-walled carbon nanotubes/CaAlg hydrogel

Letian Cheng[1], Zhilong Guo[1], Yuansheng Lin[1], Xiujuan Wei[1], Kongyin Zhao[1]* and Zhengchun Yang[2]

[1] State Key Laboratory of Separation Membrane and Membrane Processes, Tiangong University, Tianjin, 300387, China.

[2] Tianjin Key Laboratory of Film Electronic & Communication Devices, Tianjin University of Technology, Tianjin 300384, China

* Correspondence: zhaokongyin@tjpu.edu.cn (Z.K.).

1. Materials

Sodium Alginate (NaAlg) and Calcium chloride ($CaCl_2$) were purchased from Shanghai Aladdin biochemical technology Co., LTD. (Shanghai, China). Sodium chloride (NaCl) were purchased from Tianjin north sky chemical reagent Co., LTD. (Tianjin, China). Carboxyl multi-walled carbon nanotubes (CMWCNT) were obtained from Nanjing XFNANO Materials Technology Co. Ltd. (Nanjing, China). Bovine serum albumin (BSA), ovalbumin (OVA), lysozyme (Lys), bovine hemoglobin (BHb) were purchased from Lanji of Shanghai Science and Technology Development Co., LTD. (Shanghai, China). Three (hydroxymethyl) aminomethane (Tris) was obtained from Institute of biomedical engineering, Chinese academy of medical sciences. γ-aminopropyltriethoxysilane (KH550) was purchased from Tianjin United Chemical Reagent Co., LTD. (Tianjin, China). Hydrochloric acid (HCl) was purchased from Tianjin xinghua chemical reagent Co., LTD. (Tianjin, China). The Tris-HCl buffer (0.1mol/L, pH=7.4) was prepared from Tris and HCl, was used as the supporting electrolyte. Potassium chloride (KCl), potassium ferricyanide ($K_3Fe(CN)_6$) and potassium ferrocyanide ($K_4Fe(CN)_6 \cdot 3H_2O$) were all purchased from Tianjin fengchuan chemical reagent Co., LTD. (Tianjin, China).

2. Characterizations

2.1 Scanning electron micrograph (SEM)

To check the surface morphology of the membrane, select a membrane and first rinse with deionized water. After rinsing, dry the sheet in an oven and set it aside. Subsequently, the sample was

subjected to surface rotation gold spraying under vacuum conditions. In order to analyze the surface morphology obtained, a field emission Scanning electron microscope (FE-SEM: S-4800, Hitachi, Japan) was used. This technology allows for observation and comparison of NIPs membranes, MIPs membranes, and eluted MIPs membranes.

2.2 Circular dichromatic spectrum (CD) analysis

To evaluate the conformational changes in BSA solutions, we used circular dichroism to measure the conformational changes of BSA solutions before and after elution with Tris HCl buffer (0.1M, pH=7.4). To begin the experiment, we filled an appropriate amount of BSA solution into a colorimetric dish to prepare for subsequent testing.

2.3 Mechanical properties of BSA molecularly imprinted CMWCNT/CaAlg hydrogel films

In this experiment, the mechanical test was performed using a single fiber tensile tester of type LLY-06F. Film samples with a cut length of 20 mm and a width of 5 mm were stored in a beaker containing 2.5 wt.% $CaCl_2$ solution to prepare for the mechanical properties test. To perform the tensile test, the parameters of the single fiber tensile tester were set: temperature of 25 °C; speed of 10 mm/min; fracture threshold of 80%; initial spacing of 10 mm; and linear density of 1 dTex. Elongation at break was calculated as follows:

$$\lambda=100\%\times(L-L_0)/L_0 \qquad (1),$$

where L_0 and L were the initial stretching effective length (gripper spacing) and the length at stretching break of the hydrogel film, respectively.

2.4 Calculation of swelling rate

$$R_s= (W_t-W_0)/W_0\times100\% \qquad (2),$$

where W_0 and W_t were the initial mass and the mass of the films at different time points.

2.5 Adsorption properties of BSA molecularly imprinted CMWCNT/CaAlg hydrogel films

Clean vials were taken and put into 10 mL of eluent solution (pH=7.4). The cut circular diaphragms were put into the clean vials separately and eluted for 2 h. Then the diaphragms in the vials were removed and cross-linked with 2.5 wt. % $CaCl_2$ solution for about 30 min. After cross-linking, the film was put back into the eluent for 3 h. The eluted MIP and NIP films were rinsed with deionized water and placed in the corresponding labeled vials containing 10 ml of BSA solution and timed from this point on. The wavelength of the UV spectrophotometer was adjusted to 280 nm, and the absorbance of the samples was tested at this wavelength and the corresponding data traces were made. In this experiment, the absorbance at 280 nm was taken and the amount of BSA in the supernatant was calculated.

$$\text{Adsorption quantity } Q=(C_0-C_t)\times V/W \qquad (3),$$

where C_0(mg/mL) was the starting concentration of protein solution and C_t was the concentration of protein solution at a certain time, V(mL) was the volume of protein solution, and W(g) was the mass of the imprinted and non-imprinted films.

After equilibrium adsorption was reached, the equilibrium adsorption amount Q_e was recorded. The difference in protein adsorption between the BSA blotting film and the non-blotting film was compared, and the blotting efficiency IF of the protein blotting film was calculated.

$$IF=Q_{MIPs}/Q_{NIPs} \quad (4),$$

where Q_{MIPs} was the equilibrium adsorption amount of the imprinted film and Q_{NIPs} was the equilibrium adsorption amount of the non-imprinted film.

2.6 Solubilization properties of BSA molecularly imprinted CMWCNT/CaAlg hydrogel films

The CMWCNT/CaAlg hydrogel films were cut into circular pieces of approximately the same size with a hole punch, the water on the surface of the films was blotted dry with filter paper, and they were weighed and the corresponding data traces were made. Then the film was placed in a beaker containing 10 mL of 0.9 wt. % NaCl solution, and after a period of time the film was removed and the aqueous NaCl solution on the surface of the film was blotted dry with filter paper, weighed and the corresponding data tracing was done. The film weight was repeatedly weighed according to the above mentioned steps until the weight of the hydrogel film remained constant or the film swelled. The swelling rate R_s (%) was calculated as follows:

$$R_s=(W_t-W_0)/W_0\times 100\% \quad (1),$$

where W_0 and W_t were the initial mass and the mass at different points in time.

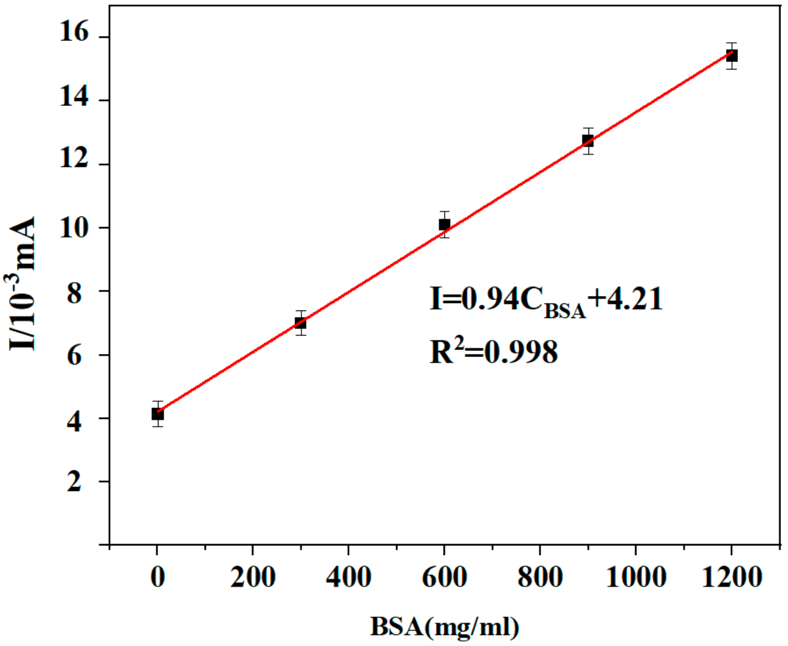

Figure S1. Calibration plot between DPV peak current and BSA concentration for MIP electrode.
